# Qualitative Release Assessment to Estimate the Likelihood of Henipavirus Entering the United Kingdom

**DOI:** 10.1371/journal.pone.0027918

**Published:** 2012-02-06

**Authors:** Emma L. Snary, Vick Ramnial, Andrew C. Breed, Ben Stephenson, Hume E. Field, Anthony R. Fooks

**Affiliations:** 1 Centre for Epidemiology and Risk Analysis, Animal Health and Veterinary Laboratories Agency, Addlestone, Surrey, United Kingdom; 2 Queensland Centre for Emerging Infectious Diseases, Biosecurity Queensland, Brisbane, Australia; 3 Wildlife Zoonoses and Vector-borne Diseases Research Group, Department of Virology, Animal Health and Veterinary Laboratories Agency, Addlestone, Surrey, United Kingdom; 4 National Consortium for Zoonosis Research, University of Liverpool, Leahurst, Chester High Road, Neston, United Kingdom; Global Viral Forecasting Initiative, United States of America

## Abstract

The genus Henipavirus includes Hendra virus (HeV) and Nipah virus (NiV), for which fruit bats (particularly those of the genus *Pteropus*) are considered to be the wildlife reservoir. The recognition of henipaviruses occurring across a wider geographic and host range suggests the possibility of the virus entering the United Kingdom (UK). To estimate the likelihood of henipaviruses entering the UK, a qualitative release assessment was undertaken. To facilitate the release assessment, the world was divided into four zones according to location of outbreaks of henipaviruses, isolation of henipaviruses, proximity to other countries where incidents of henipaviruses have occurred and the distribution of *Pteropus* spp. fruit bats. From this release assessment, the key findings are that the importation of fruit from Zone 1 and 2 and bat bushmeat from Zone 1 each have a *Low* annual probability of release of henipaviruses into the UK. Similarly, the importation of bat meat from Zone 2, horses and companion animals from Zone 1 and people travelling from Zone 1 and entering the UK was estimated to pose a *Very Low* probability of release. The annual probability of release for all other release routes was assessed to be *Negligible*. It is recommended that the release assessment be periodically re-assessed to reflect changes in knowledge and circumstances over time.

## Introduction

The genus Henipavirus includes Hendra virus (HeV) and Nipah virus (NiV), which have been associated with disease in horses and pigs, respectively. From 1994, 26 outbreaks of henipaviruses have occurred in Australia, Malaysia, Singapore, India and Bangladesh [1], [2], with consequent trade implications in Oceania and Asia [3]. Even more significantly, both viruses are zoonotic, and have caused substantial mortality in humans (case fatality rate for HeV is 57% [4] and variable for NiV ranging from 9% in Singapore to 100% in Nadia, India [1]). Therefore both HeV and NiV are major public health concerns.

HeV was first discovered in September 1994 following a sudden outbreak of an acute respiratory syndrome in thoroughbred horses in a racing stable in Brisbane, Australia. Thirteen out of 20 infected horses died and a stable-hand and trainer were infected, the latter fatally [5]. Since 1994 there have been 14 HeV outbreaks that have resulted in horse fatalities [5], [6] and to the time of writing there have been a total of 7 human cases (4 deaths), all of which were associated with contact with horses [7]. In order to contain the outbreaks, procedures were implemented, such as movement restrictions; destruction and sanitary disposal of horses shown to be infected (by presence of antibodies); disinfection of the environment and quarantine of in-contact animals until they have been shown to be free from HeV (by repeated serological tests) [8]. Serological evidence of HeV infection was identified in fruit bats of the genus *Pteropus* (commonly known as flying-foxes). All four species of flying-fox that occur in mainland Australia (*Pteropus alecto*, *Pteropus poliocephalus*, *Pteropus scapulatus* and *Pteropus conspicillatus*) were subsequently identified as reservoir hosts for HeV [9].

Four years later, in late September 1998, a novel disease outbreak in pigs in peninsular Malaysia and an associated outbreak of typically severe febrile encephalitis in humans was identified. A Hendra-like virus, subsequently named Nipah Virus was identified as the etiologic agent [10]. Between September 1998 and May 1999, 265 encephalitis patients, which included 105 deaths [3], were reported in three states of Malaysia [11]. Most patients with Nipah encephalitis in Malaysia were pig farmers. The virus isolates obtained from both human patients and sick pigs showed identical nucleotide sequence [11] and therefore pigs were implicated as the primary source of human infection. Over one million pigs were culled to control the outbreak [10]. Malaysian bat species were prioritised for surveillance to identify the reservoir of NiV subsequent to the discovery of fruit bats being the likely reservoir for HeV in Australia. Blood and tissue samples were collected and *Pteropus vampyrus* and *Pteropus hypomelanus* were identified as likely natural reservoir hosts [9]. Outbreaks of NiV have since occurred in Bangladesh where, as in Malaysia and Australia, fruit bats of the genus *Pteropus* (*Pteropus giganteus*) were identified as a putative natural reservoir [12]. Horizontal human transmission was also documented in the 2004 NiV outbreak in the Faridpur district in Bangladesh [13], [14] and also in Siliguri India, which is close to affected areas in Bangladesh [15]. In Singapore workers at pig abattoirs in which Malaysian pigs were slaughtered were infected with NiV [16]. In Malaysia 7 pig abattoir workers showed antibody against NiV and had therefore been exposed to the virus [11].

**Figure 1:**
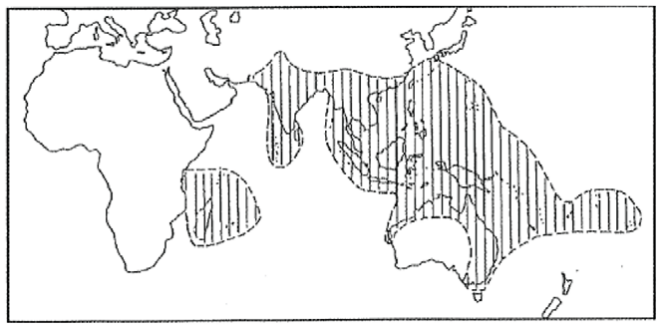
World distribution of fruit bats of genus *Pteropus* [**17**].

Overlapping distributions of only three species of fruit bats are needed to form a continuous link between the east coast of Australia and Pakistan. The geographic distribution of pteropid bats is shown in Figure 1 [17]. The species of fruit bats that are believed to be important in the transmission of henipaviruses because of their implication in outbreaks include: (i) *P. alecto*, *P. poliocephalus*, *P. scapulatus* and *P. conspicillatus* [18]; (ii) *P. vampyrus* [9], [19], [20]; (iii) *P. hypomelanus* [9], [19], [20]; and (iv) *P. giganteus* [12], [21], [22]. Currently, data on the prevalence of henipaviruses in fruit bats are limited. Most studies have been undertaken to estimate the seroprevalence in various species of fruit bats and the results are provided in Table 1. The data from Table 1 were obtained from non-random sampling of the fruit bats and show a range in seroprevalence from 1% (*P. rufus*, Madagascar [23]) to 63% (*P. vampyrus*, Malaysia [19]). More recently, HeV and NiV antibodies have been detected in non-pteropid bats in Madagascar [23] and China [24], and both antibodies to and viral RNA of henipaviruses have been detected in Ghana [25], [26]. Fruit bats have not developed clinical disease when experimentally infected with henipaviruses [27], [28].

**Table 1:** Seroprevalence of henipaviruses (HeV and NiV) in fruit bats.

Species of bat	Location of study	Sero-acting virus	Number tested	Number positive	Sero-prevalence (95% CI, where given)	Reference
*P. alecto, P. poliocephalus, P. scapulatus, P. conspicillatus*	Australia	HeV	1,043	-	47%	Field *et al*., 2001 [3]
*P. vampyrus*	Indonesia	HeV	79	19	24%	Sendow *et al*., 2006 [22]
	Indonesia	NiV	84	32	38%	Sendow *et al*., 2006 [22]
	Malaysia	NiV	56	35	63%	Shirai *et al*., 2007 [19]
	Malaysia	NiV	29	5	17%	Johara *et al*., 2001 [29]
	Thailand	NiV	39	1	2.6%	Wacharapluesadee *et al*., 2005 [20]
*P. hypomelanus*	Malaysia	NiV	102	18	18%	Shirai *et al*., 2007 [19]
	Malaysia	NiV	35	11	31%	Johara *et al*., 2001 [29]
	Thailand	NiV	26	4	15.4%	Wacharapluesadee *et al*., 2005 [20]
*P. giganteus*	India	HeV	39	11	28%	Epstein *et al*., 2008 [21]
	India	NiV	39	20	54%	Epstein *et al*., 2008 [21]
*P. lylei*	Thailand	NiV	813	76	9.3%	Wacharapluesadee *et al*., 2005 [20]
*E. helvum*	Ghana	HeV	59	13	22% (11–33)	Hayman *et al*., [26]
	Ghana	NiV	59	23	39% (27–51)	Hayman *et al*., [26]
*E. dupreanum*	Madagascar	HeV	73	11	15%	Iehle *et al*., 2007 [23]
	Madagascar	NiV	73	14	19%	Iehle *et al*., 2007 [23]
*P. rufus*	Madagascar	HeV	349	2	1%	Iehle *et al*., 2007 [23]
	Madagascar	NiV	349	6	2%	Iehle *et al*., 2007 [23]

HeV and NiV have an extended host range. During the 1998/1999 NiV outbreak in peninsular Malaysia infection occurred in domestic dogs and cats that were exposed to infected pigs [29], [30]. At a later date (2004) cats at the site of the outbreak and near to a bat colony with antibodies to NiV were not infected [31]. When experimentally infected with either Hendra or Nipah viruses cats develop severe clinical disease [32], [33], [34]. HeV has been experimentally shown to infect guinea pigs and pigs [27], [31]. Although the clinical signs in pigs infected with HeV seem to be more severe than those for NiV [35] this may be due to the high experimental dose. Infection of humans is usually from an amplifier host, such as from pigs for NiV [10] or horses for HeV [5], however human NiV infection from fruit bats has been reported [12], [36].

The mode of transmission to humans and animals is uncertain. However, it is thought to be via close contact with contaminated tissue or body fluids from infected animals or eating foodstuffs contaminated with fruit or fruit pulp disgorged by fruit bats. NiV was isolated from partially eaten fruit in peninsular Malaysia [20] and the ingestion of contaminated date palm sap has been implicated in NiV outbreaks in humans in Bangladesh [37].

The ability of henipaviruses to produce disease that causes significant mortality in humans has made this emerging viral infection a serious public health concern. To estimate the likelihood of henipaviruses entering the UK, a qualitative release assessment was undertaken. Using the World Organisation for Animal Health (OIE) methodology for import risk analysis, the release assessment describes “the biological pathways(s) necessary for an importation activity to ‘release’ (that is, introduce) pathogenic agents into a particular environment, and estimating the probability of that complete process occurring…” [38]. Therefore, the risk question was defined as ‘*what is the annual probability of henipaviruses being released into the UK via imported animals, imported foodstuffs and humans?*’ Due to the genetic and pathogenic similarity of HeV and NiV, combined with their high level of cross reactivity and similar disease ecologies, the henipaviruses are considered at the generic level for this study.

## Methods

**Figure 2:**
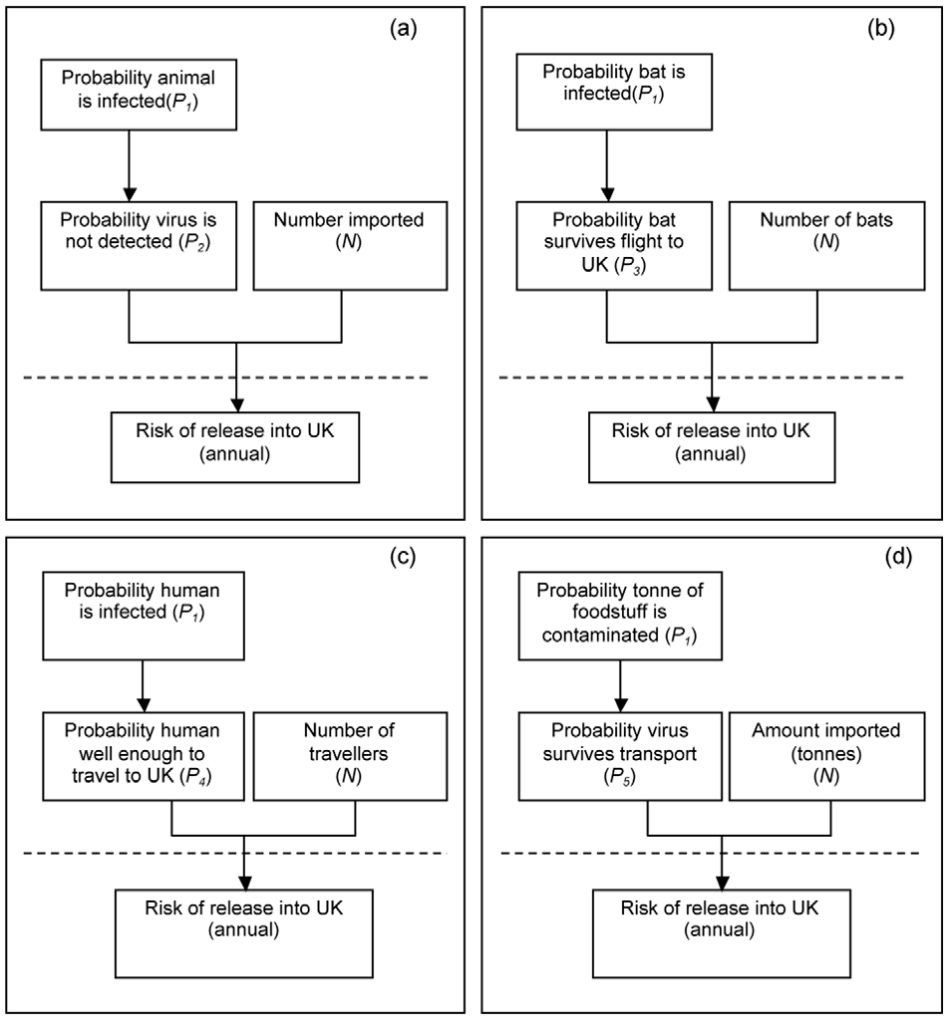
Release pathways for henipaviruses to be released into the UK via (a) imported bats (all species), pigs, horses and companion animals; (b) “natural importation” of insectivorous bats; (c) via human travel; (d) via a tonne of imported foodstuffs.

The routes considered within the release assessment are: imported bats (all species) (Figure 2a); insectivorous bats flying into the UK (Figure 2b); imported pigs, horses and companion animals (Figure 2a); humans (Figure 2c) and imported food stuffs, which includes pigmeat, fruit, fruit juices and bat bushmeat (Figure 2d). Using the pathways given in Figure 2, a release assessment was developed using the risk analysis guidelines developed by the World Organisation for Animal Health [39]. In order to standardise the different qualitative probabilities assigned within the release assessment and to increase transparency, each probability has been defined using definitions used by the European Food Safety Authority [40], see Table 2.

**Table 2:** Definitions of risk [40].

Probability Category	Interpretation
*Very High*	Event occurs almost certainly
*High*	Event occurs very often
*Medium*	Event occurs regularly
*Low* *	Event is rare but does occur
*Very Low* *	Event is rare but cannot be excluded
*Negligible*	Event is so rare that it does not merit to be considered

*Note: it is possible that the event is occurring but is not detected by current surveillance schemes/methods.

The probability of henipaviruses being released into the UK (*R*) for the pathways in Figure 2 can be assessed by combining the number that is imported annually (*N*) with the results from the probability pathway (*P*) which assesses the probability per animal, human or tonne of foodstuff. *P* is estimated by considering the multiplicative outcome of two probabilities *P_1_* and *P_2_*, *P_3_*, *P_4_* or *P_5_* where *P_1_* is defined as the probability that a source is infected or contaminated. The probabilities *P_2_*, *P_3_*, *P_4_* or *P_5_* provide the conditional probability given that the animal or human is infected or foodstuff contaminated henipavirus is introduced to the UK. Therefore *P_2_* is the probability that an animal that is infected with henipavirus is not detected on entering the UK; *P_3_* is the probability that an infected bat survives flight to the UK; *P_4_* is the probability that a human infected with henipavirus is well enough to travel and, finally, *P_5_* the probability that the virus present on or in a foodstuff survives transport to the UK. The qualitative estimates for the probabilities *P_1_* and *P_2_*, *P_3_*, *P_4_* or *P_5_* are combined using the matrix approach as described by Gale *et al.* 2009 [41], which is used to describe probabilities that are multiplicative. Using this matrix, the maximum probability (*P*) is the minimum of the multiplied probabilities. The combination of *P* and *N* is not multiplicative and is considered on a case-by-case basis.

### 1 Assessing the probabilities per individual animal, human and tonne of foodstuff (*P*)

For each release pathway, the probability of importing henipaviruses per animal, per human or per tonne of foodstuff is estimated (*P*) (Table S1). Outside the UK, the transmission routes between different animal species, food stuffs and humans are not considered. The following sub-sections summarise the estimation (in qualitative terms) of the probabilities *P_i_, i = 1,…, 5*.

#### 1.1 Fruit bats

**Figure 3:**
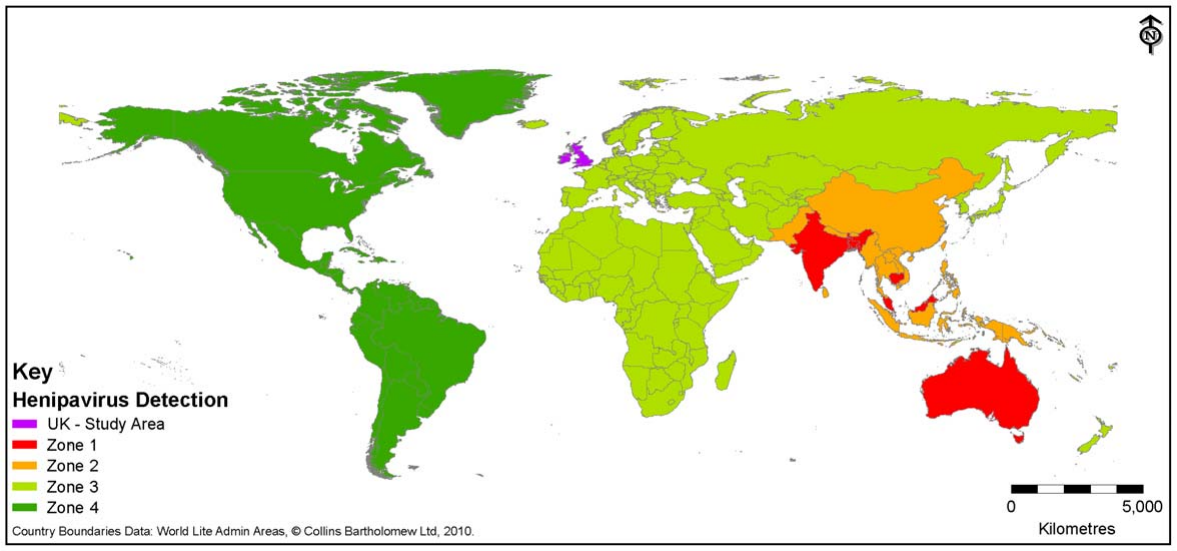
Identification of risk zones for henipavirus. Zone 1: countries with outbreaks of henipaviruses or where henipaviruses have been isolated from fruit bats. Zone 2: country that borders a Zone 1 country and *Pteropus* fruit bats are distributed in the country. Zone 3 indicates all other countries in Eurasia, and Africa and Australasia, which includes countries that have detected antibodies to henipaviruses in fruit bats. Zone 4: North and South America, which share no bat species with the “Old World” and no henipaviruses have been isolated or antibodies detected.

Information on the location of outbreaks of henipaviruses, virus isolation and the geographic distribution of fruit bats (genus *Pteropus*) were combined to divide the world into four zones (Figure 3). Zone 1 includes countries with outbreaks of henipaviruses or where henipaviruses have been isolated from fruit bats and is therefore assumed to present a higher degree of interaction, or potential interaction, between infected fruit bats and humans or domestic animals (Australia, Bangladesh, Cambodia, India, Malaysia). In Singapore, the human cases were abattoir workers who developed the disease at the same time of the Malaysian outbreak. In the abattoir, 80% of pigs slaughtered were from Malaysia and it was therefore concluded that this was the source of the outbreak [42]. Given the different epidemiology in Singapore, this country is not included within Zone 1. No outbreaks have occurred in Cambodia but NiV has been isolated from fruit bats [43]. A Zone 2 country borders a Zone 1 country and fruit bats (specifically *Pteropus*) are distributed in the country (Bhutan, China, Indonesia, Laos, Myanmar, Nepal, Pakistan, Papua New Guinea, Philippines, Sri Lanka, Taiwan, Thailand, Vietnam). Zone 3 includes all other countries in Eurasia, Africa and Australasia that have detected antibodies that cross-react with henipaviruses in fruit bat species. Finally North and South America are classified as Zone 4 as they share no bat species with the “Old World” and there is no evidence for henipaviruses in people or animals. Consequently, Zone 4 is not considered further within this assessment as the probability of release is deemed to be negligible. Using the information available and the definitions provided in Table 2 it is estimated that the probability of a fruit bat being infected with henipavirus (*P_1_*) in Zone 1 is *Medium* because outbreaks have occurred due to direct or indirect contact with infected fruit bats. Although no outbreaks have occurred in Zone 2, countries within this zone border a Zone 1 country and *Pteropus* fruit bats, that can travel considerable distances, are present and therefore *P_1_* is assessed to be *Low*. For Zone 3, the geographic distribution of fruit bats and distance that fruit bats can travel suggest that there is a *Very Low* probability of fruit bats being infected.

Fruit bats imported to the UK must comply with the Rabies Import Order and spend 6 months in quarantine, excluding vampire bats which must spend their lives in quarantine. Currently, no tests for henipaviruses are undertaken for bats imported to zoos. Fruit bats do not develop clinical disease when experimentally infected with henipaviruses [27], [28]. It is therefore assumed, due to the combination of both a lack of diagnostic screening and the absence of clinical signs, that detection of henipavirus is unlikely. In addition, there is evidence for latent infection and recrudescence in bats [44] which may also increase the likelihood of not detecting the virus. Therefore the probability of the virus not being detected in fruit bats (*P_2_*) is assessed to be *High*.

#### 1.2 Insectivorous bats

There is evidence to suggest that insectivorous bats can be infected with henipaviruses; antibodies for NiV were detected in *Scotophilus kuhli* in Malaysia [29] and in *Myotis* species in China [24]. *Myotis daubentonii* is distributed across Europe and Asia, including the UK [45]. No surveys for henipaviruses in insectivorous bats in the proximity to the UK have been reported so knowledge of the presence or absence of infection is lacking and no outbreaks have occurred in the European Union (EU). Further, it is reasonable to assume that an infected insectivorous bat is more likely to be located where infected fruit bats are located (e.g. Asia, Oceania). Given that fruit bats are the reservoir host and hence the need for direct or indirect transmission to occur, insectivorous bats are predicted to have a *Low* probability of infection in Zone 1. Similarly, a *Very Low* probability of infection is estimated for Zone 2 and a *Negligible* probability of infection in Zone 3.

For imported bats, tests for henipaviruses are not undertaken prior to entry to the UK. Very little is known on whether infected insectivorous bats show clinical disease. However, work by both Johara *et al.* [29] and Li *et al.* [24] detected antibodies to NiV or a closely related virus in apparently healthy insectivorous bats, which indicates that infection in insectivorous bats is at least sometimes non-fatal, and possibly sub-clinical. The incubation period of HeV and NiV in insectivorous bats is unknown. Given the above, it is assumed that *P_2_* is *High*, although there is a high level of uncertainty associated with this estimate of probability.

It is improbable that an insectivorous bat from Asia or Oceania would survive a journey to the UK due to the large distance to travel and therefore only Zone 3 is considered for the release of henipaviruses via insectivorous bats flying to the UK. Similar to the above, the probability of a bat surviving such a journey will be related to the likelihood and severity of clinical signs, it is therefore predicted that *P_3_* is *High*.

#### 1.3 Companion animals

Companion animals may enter the UK from countries in which henipaviruses are present via 6-month quarantine or, if eligible, the Pet Travel Scheme (PETS) which has a 6 month waiting period [46]. From Zone 1, only cats and dogs from Australia and Malaysia may enter via the PETS scheme as a listed third country. Likewise, Zone 2 animals from all but one country (Taiwan) will undergo quarantine procedures. Companion animal movements from Zone 3 countries will be under PETS (EU Member States (MSs) or listed third country) or 6 month quarantine. During the 6 month waiting period for PETS the animal is not confined and thus there is an increased likelihood (compared to quarantine) of an animal being exposed to henipaviruses if infection is present in the country of origin.

In addition to PETS, there is a further restriction on the import of companion animals from peninsular Malaysia. Cat or dog movement is prohibited unless health certification is provided by the Malaysian Veterinary Authority to confirm that the animal: (i) has had no contact with pigs during at least the 60 days prior to export; (ii) has not been resident on holdings where during the past 60 days any case of Nipah disease has been confirmed; and (iii) has had a negative result to an ELISA for Nipah antibody on a sample of blood taken within 10 days of export undertaken in a laboratory approved by the Veterinary Authority [47]. Furthermore, cats that have been resident in Australia must have a certificate from the Australian Veterinary Authority confirming that at no time have they been on a holding where HeV has been confirmed during the 60 days prior to departure [47].

In assessing the probability of a companion animal being infected, it is considered that natural HeV infection in cats has never been reported. Given that fruit bats are the reservoir host and hence the necessity of direct or indirect transmission to occur for cats or dogs to be infected, it is assumed that cats and dogs have a much lower prevalence of infection than fruit bats, although there is uncertainty here as there is a lack of information on the degree of contact between cats and dogs and fruit bats. Additional restrictions in Australia and Malaysia will also reduce the probability of a cat or dog intending to travel to the UK being infected. It is therefore estimated that the probability of a cat or dog that is intending to travel to the UK from Zone 1 being infected with henipavirus is *Very Low* and *Negligible* for Zones 2 and 3, respectively.

The incubation period for HeV and NiV in experimentally infected cats is 4–8 days [34] and 6–8 days [32], respectively. The incubation period for NiV in dogs is unknown and therefore is assumed to be similar to cats. Clinical signs for henipaviruses in cats are fever, increased respiratory rates, followed by severe illness and death within 24 hours. Clinical signs in dogs for NiV include fever, respiratory distress, conjunctivitis and severe cases result in death. Mills *et al.* [48] reported that NiV infection was detected, by immunohistochemical examination, of 2 dogs (1 dead; 1 dying) that were in the epidemic area of the NiV outbreak in peninsular Malaysia. Consequently, it is likely that an infected incubating companion animal will display signs when entering the UK via quarantine or PETS as the schemes' waiting period is substantially longer than the incubation period. As a consequence it is predicted that the probability of not detecting infection (*P_2_*) is *Very Low* for all three zones, but it is noted however that illegal movements of animals – not subject to PETS or Quarantine – will have a higher probability.

#### 1.4 Pigs

NiV infection in pigs is highly transmittable and as described above, pigs were implicated as the primary source of human infection in the outbreak in Malaysia in 1998–1999 [11]. It is believed that the establishment of pig farms within the range of the natural host and the planting of fruit trees near these farms led to the initial introduction of henipaviruses into the pig population [49]. The maintenance of high densities of pigs led to the rapid dissemination of the infection within local pig populations, and the transport of pigs to other areas for commerce led to the rapid spread of disease in pigs in southern Malaysia and Singapore [3].

Natural HeV infection has never been reported in pigs. In 500 serum samples collected from 100 swine herds in Queensland (Australia) anti-HeV antibodies were not detected. Given the susceptibility of pigs to HeV under experimental conditions, the possibility of pigs being naturally infected with HeV in Australia cannot be completely dismissed, but is considered to be at a *Very Low* probability. This is taken into account when accessing the likelihood of pigs and also pig meat products being infected or contaminated, respectively, with henipavirus.

Given the previous outbreaks in Bangladesh, India and Malaysia and the necessary transmission of the virus from fruit bats it is predicted that there is a *Low* probability of a pig from these countries being infected, but a *Very Low* probability for Australia and Cambodia. Overall the probability of a pig from Zones 1, 2 and 3 being infected (*P_1_*) is estimated to be *Low*, *Very Low* and *Negligible*, respectively.

NiV has an incubation period of 4–14 days in pigs. Clinical signs in pigs include fever, respiratory distress and neurological signs such as trembling, twitching and muscle spasms [50]. Tests for henipaviruses are not undertaken on pigs entering the UK. Imports of pigs from specified third countries must comply with the animal health conditions laid down in the Community legislation and be accompanied by a health certificate, signed by an official veterinarian of the Veterinary Authority in the country of origin [51]. A previous risk assessment estimated shipping times to the UK from different regions of the world [52], which are also used here. The estimated minimum shipping time from Oceania is 19.5 days; 12.6 days from Southern Asia; 17.1 days from South Eastern Asia; 16.4 days from Eastern Asia and 5.2 days from West Africa. Therefore, it is possible for a pig that is incubating the disease to enter the UK if imported from Zone 1 particularly if imported from Southern Asia (12.6 days) and therefore it is estimated that the probability of the infection not being detected (*P_2_*) is *Low*. Zone 2 countries are in Southern Asia, Eastern Asia and South East Asia, for which the minimum journey time ranges from 12.6 days to 17.1 days therefore, as above, *P_2_* is estimated to be *Low*. A journey time from Zone 3 will vary greatly and may be via sea or land. The journey time is therefore assumed to range from 1 day (overland travel within Europe) to 11.4 days (Southern Africa) and consequently it is estimated that *P_2_* is *Medium* for Zone 3.

#### 1.5 Horses

HeV was discovered in thoroughbred horses in a racing stable in Brisbane, Australia, in 1994. Horses displaying clinical signs can survive infection, and both field observations and laboratory transmission experiments have shown that although horizontal transmission can occur, it does not occur readily. Infected horses can also be asymptomatic or have mild clinical signs [3]. Given the occurrence of outbreaks in Australia and the potential possibility of cases of HeV in other Zone 1 countries (although never reported) it is assumed the probability of a horse in Zone 1 being infected is likely to be *Low*. Again, as with previous release routes, this takes into account the necessity for the virus to be transmitted to horses from the reservoir hosts, fruit bats. For Zone 2 and Zone 3, it is estimated that the probability *P_1_* is *Very Low* and *Negligible*, respectively.

Horses are imported to the UK from Zones 1, 2 and 3; however tests for henipaviruses are not undertaken prior to entry although veterinary checks will be undertaken. The incubation period of HeV in horses ranges from 4 to 16 days, but is typically 8–11 days [27], [53], [54]. Clinical signs of HeV in horses include depression, ataxia, tachycardia, fever and death for acutely affected horses with severe respiratory distress [27]. Using the information given above for minimum shipping times it can be concluded that it is possible for a horse that is incubating the disease to enter the UK if travelling by sea. However, it is also noted that due to the high value placed on many of the horses entering the UK (e.g. for show jumping; racing) horses may also enter by land or by ‘plane. Consequently the minimum journey time is assumed to be one day for all zones and therefore the probability of not detecting a horse infected with henipavirus (*P_2_*) is assessed to be *High*.

#### 1.6 Humans

Evidence from past outbreaks has shown that humans can become infected with henipaviruses through contact with a domestic animal amplifying host (pigs [10], horses [5]), humans [13], [14], [15] and from direct contact with fruit bats [12], [36]. In relation to the probability of a human being infected (*P_1_*) it is considered that *P_1_* will be *Very Low* in Zone 1 and *Negligible* in Zones 2 and 3. This takes into account the number of reported cases within each zone, the total human population sizes and also an assumption that this probability is likely to be less than *P_1_* for a pig or horse.

In humans, the incubation period is generally between 4–45 days for NiV [50] and 5–21 days for HeV [6], [55]. Human infections of NiV range from asymptomatic to fatal encephalitis. Initial symptoms in humans include myalgia, fever and the disease may progress to encephalitis with drowsiness, disorientation, convulsions, coma and severe cases result in death [50]. Symptoms for HeV range from mild influenza-like illness to fatal respiratory or neurological disease.

Humans are not tested for henipaviruses and journeys to the UK will typically be less than 1 day. Therefore, a human who is incubating the disease could introduce henipaviruses to the UK if well enough to travel and as a consequence of this the probability *P_4_* is estimated to be *High*.

#### 1.7 Foodstuffs

Foodstuffs that could plausibly be contaminated with henipaviruses include fruit, date palm sap, fruit juice, pork products and bat bushmeat. Fruit bats mainly feed on fruit and thus there is an increased likelihood of fruit being contaminated with urine and saliva in areas where fruit bats roost and feed (NiV has been isolated from partially eaten fruit [56]). There is evidence of food-borne transmission, for example date palm sap in Bangladesh has been identified as a risk factor for human infection [36]; however it cannot be ascertained from import data if this particular product is imported into the UK and, if so, how much is imported. For this reason, date palm sap is not considered further. Given the high degree of contact between fruit bats and fruit there is a possibility of fruit being contaminated with saliva from fruit bats infected with henipaviruses. Therefore it is predicted that the probability that a tonne of fruit or fruit juice is contaminated (*P_1_*) is *Low* for Zone 1. Likewise, for Zone 2 and Zone 3 it is assessed to be *Very Low* and *Negligible*, respectively.

Pork products may also be a source for henipaviruses to be released into the UK if pigs are infected. No information was obtained on the presence of NiV in meat from infected pigs and therefore, as a worst case scenario, it is assumed that the meat will be contaminated. Members of the *Paramyxoviridae*, although enveloped viruses, are heat-sensitive. Therefore using this information it is assumed that cooked and cured products will have a lower probability of contamination than raw pork products. It is probable that freezing meat contaminated with henipaviruses, with subsequent thawing, would have a minimal effect in reducing the viral load. It is therefore assumed that the probability associated with frozen pork products (*P_1_*) is equivalent to fresh, chilled pork products, which are predicted to be *Low* in Zone 1; *Very Low* in Zone 2 and *Negligible* in Zone 3. Due to the processes undertaken (e.g. cooking, curing) the probability per tonne of processed pork (*P_1_*) is estimated to be *Very Low* in Zone 1, but *Negligible* for Zones 2 and 3.

Another potential route for the release of henipaviruses into the UK is via illegally imported bushmeat, particularly bat meat, which is consumed in many countries in Asia and Africa [26]. For bushmeat being illegally imported into the UK, a tonne would consist of a large number of bats, not all of which will be of the genus *Pteropus*. However, because these types of bats are larger than many other species it is possible that these may be preferentially selected for bushmeat trade. As a tonne of bushmeat would consist of a large number of bats and therefore given the *Medium* probability of infection in Zone 1 for an individual bat, it is predicted that a tonne of bushmeat originating from Zone 1 would have a *High* probability of contamination, *P_1_*. For the same reasons as described above it is predicted that the corresponding probability of a tonne of Zone 2 bushmeat containing henipavirus is *Medium*. Given the *Very Low* probability of Zone 3 fruit bats being infected, it is assumed that the probability of contamination per tonne of bushmeat is *Very Low*.

Tests for henipaviruses are not undertaken for legal foodstuffs or, by definition, illegal foodstuffs prior to entry into the UK. However a foodstuff entering the UK could only be contaminated if the virus survives the journey. Import data available from H M Revenue and Customs suggest that 90–100% of non-EU fruit commodity enters the UK by sea, the exception mostly being berries (43%). From the EU, 88% arrives by sea, 8% by road and only 0.03% by air. Travel by air would most likely be reserved for highly perishable, non-bulky, high value commodities. Although the commodity codes make it difficult to know exactly what fruit was imported, many of the commodity codes provide an indication of fruit that is likely to be imported via air into the UK from Zone 1. Pig meat (fresh, frozen or processed) is rarely imported via air freight. However, bushmeat may enter the UK via air passengers luggage and therefore travel time may be less than 1 day. Shipping times from different regions in the world are provided in Section 2.1.4.

Fogarty *et al.* [57] reported that at 37°C, henipaviruses are virtually inactivated in 1 day in pH-neutral bat urine from the genus *Pteropus*. However, repeating the experiment with urine at its natural pH of 2, inactivation was significantly quicker (less than 30 mins at both 22°C and 37°C). At 22°C, henipaviruses can survive for more than 4 days in pH-neutral bat urine. The same study also considered survival of henipaviruses in fruit juice and on mango flesh. On mango flesh, survival time ranges from 2 hours to more than 2 days, however desiccation reduces the survival time of henipaviruses to less than 2 hours. In acidic fruit juice, inactivation took less than 4 days. No information was reported on the survival of henipaviruses on pork products or bat meat; although it is known that the pH of meat ranges from 5.2–7 and therefore slightly acidic. Additional quantitative studies on the survival of henipaviruses on different foodstuffs are required.

Using the information given above for survival of HeV and NiV and transport times from different regions of the world, it is predicted that there is a *Negligible* probability of henipavirus surviving on pig meat (fresh, frozen or processed) imported from Zone 1 or Zone 2 (*P_5_*). From Zone 3, which includes European countries that are clearly much nearer the UK, it is assumed that there is a *Medium* probability of henipavirus surviving the journey. For fruit, it is unlikely that the virus will survive for the duration of transport to the UK if being shipped; however up to 10% of some fruit commodities will not enter by ship but by air and therefore significantly reducing the transport time and having a higher probability of survival due to a decreased temperature during the transport as airfreight. Given this, it is predicted that the probability of henipavirus surviving in a tonne of fruit is *Low* for both Zones 1 and 2. As was previously the case, travel times will be shorter from Zone 3 and therefore the probability of the virus surviving (*P_5_*) is estimated to be *Medium*. Similarly for fruit juice the probability *P_5_* is estimated to be *Medium* for Zone 3, but *Negligible* for Zones 1 and 2 due to the assumption that juice imported from Zones 1 and 2 will enter the UK via ship. Finally, for bushmeat the worst case scenario was taken, which is that the meat will enter via air (i.e. passengers bringing the meat in their personal luggage) and therefore it was assumed that the travel time is 1 day. Consequently, the probability of henipaviruses surviving (*P_5_*) is assessed to be *High* for all Zones.

### 2 Assessing the number of imports (*N*)

Data were collected for the number of animals (bats, companion animals, pigs, horses), the number of humans travelling to the UK [58], and the amount of foodstuffs (tonnes) imported in 2008 (see Table S1). Trade statistics were supplied by Defra (from H. M. Revenue and Customs datasets) or Animal Health (bats and companion animal trade information only). If quantitative data were not directly available, a qualitative estimate of the amount of imports is provided. It is important to note that these qualitative quantities do not correspond to those provided in Table 2, which relates to probabilities, and that the assigned levels of importation are only appropriate for 2008 and therefore may change (significantly) in future years.

The number of horses imported from Zone 3 is highly uncertain due to a £260,000 (approximately $416,500**)** threshold that is applied to horses coming from EU MSs, i.e. only horses above this value are reported. From the EU, 2876 horses were recorded as entering the UK, which is assessed to be a *Low* level of importation. In order to adjust for the under-reporting, this qualitative estimate is increased to *Medium* as many horses from Zone 3 are likely to be imported that are beneath this high threshold value. There is also uncertainty associated with the data for the amount of fruit imported into the UK. The two commodities recorded are fresh or dried fruit and prepared or preserved fruit. Clearly, the process of drying or preserving fruit will have an impact on the probability of the virus surviving – fresh fruit and fruit preparations (i.e. without any processing) are the main commodities of interest but it is not possible to distinguish these types. Therefore all fruit imports are included in the release assessment, which provides a worst case scenario.

Chaber *et al.* 2010 [59] estimate that 3,287 tonnes of meat and fish are illegally imported via Paris Roissy-Charles de Gaulle airport from Africa; 273 tonnes (8%) is bushmeat. No bat meat was seized. However the study took place over a brief period (1 week) and searched a relatively small number of passengers. A VLA risk assessment for illegal meat [52] estimated that between 4,398 and 28,626 tonnes per year is illegally imported into Great Britain (GB), which compared to legally imported meat is very small. Eastern Asia, Near and Middle East, Eastern Europe, Southern Africa and Western Africa were the regions with the highest rates of illegal meat flow. However, information was not obtained on the proportion of illegal meat that is bat meat. Considering the total amount of illegal meat imported from each zone relative to the quantities of foodstuffs imported via legal routes, it is concluded that Zone 1 has a *Very Low* level of illegal imports; Zone 2 has a *Low* level and Zone 3 has a *Medium* level. Mickleburgh *et al.* [60] reviewed the consumption of bat meat in many countries, including the frequency of consumption and the amount that is traded, and these data are used to provide an indication of the desire of visitors or UK tourists to bring bat bushmeat to the UK. For example, in Cambodia and Malaysia (Zone 1), bat meat is regularly eaten and there is internal trading of the product. Given this information, and the fact that the amount imported is unlikely to be negligible, it is predicted that the number of tonnes of bat bushmeat brought into the UK from Zone 1 will be *Very Low*. Likewise, for Zone 2, the tonnage of illegal bat meat is estimated to be *Very Low* due to many countries in this zone (e.g. China, Laos, Thailand, etc.) regularly consuming and trading bat bushmeat. Finally, it is estimated that the amount of bat meat brought into the UK from Zone 3 is also *Very Low*. This is due to the intensity of consumption and trade in Sub-Saharan Africa, compared to South East Asia and East Asia, being lower which is also alluded to by Jenkins & Racey, 2008 [61].

## Results

Combining the qualitative estimates for the probabilities *P_1_* and *P_2_*, *P_3_*, *P_4_* or *P_5_* the animal or human release route with the highest probability, per individual, (*P*) was assessed to be fruit bats imported from Zone 1, which had a *Medium* probability of being infected at the point of entry to the UK (Table S1). This is due to the medium probability of a fruit bat being infected; combined with a high probability of non-detection and survival during the importation process. Likewise, for foodstuffs, the release route with the highest probability was a tonne of illegally imported bat bushmeat from Zone 1, which had a *High* probability of being contaminated. This was attributable to the *High* probability of a tonne of bushmeat containing a fruit bat that had been infected with a henipavirus and the high probability of virus survival if bushmeat enters the UK via personal baggage on an aeroplane.

On a per unit basis, many Zone 3 routes were assessed to have a *Negligible* probability of release due to a *Negligible* probability of infection or contamination (*P_1_*). However due to long shipping times many products imported from Zones 1 & 2 were also assigned a *Negligible* probability of contamination (pork products & fruit juice) at the point of entry to the UK. Therefore, per unit, the routes with a non-negligible probability of release (*P*) are fruit bats (all zones), imported insectivorous bats (Zones 1 & 2), companion animas (Zone 1), pigs (Zone 1 & 2), horses (Zone 1 & 2), humans (Zone 1), fruit (Zone 1 & 2) and bat bushmeat (all zones).

In relation to the annual probability of release, the number of imports (*N*) and *P* are combined using a non-matrix approach, i.e. assessing each combination of *N* and *P* on a case-by-case basis. It is assumed that if the number of imports (*N*) is negligible, that the probability of release is also negligible. Many of the release routes were predicted to have a non-negligible annual probability of release, but these were assessed to be *Low* (importation of fruit from Zone 1 and 2 and bat bushmeat from Zone 1) or *Very Low* (importation of bat meat from Zone 2, horses and companion animals from Zone 1 and people travelling from Zone 1).

## Discussion

The main aim of this release assessment was to determine the different likelihoods of henipavirus introduction associated with different release routes and areas of the world. As expected, many of the non-negligible probabilities are associated with Zone 1, which includes countries with recent outbreaks of henipaviruses or where henipaviruses have been isolated. The possibility of infected humans entering the UK may be of public health concern especially as horizontal transmission of NiV was documented in Bangladesh [13] and a high case fatality rate reported. Similarly, there has been evidence of direct transmission of HeV from horses to humans [62]. Although a large number of release routes have been considered, these are not exhaustive. For example, due to a lack of knowledge on the amount of date palm sap imported into the UK it was not possible to assess the probability of release from this food product.

For many of the imports (animals and foodstuffs), no products were imported and therefore *Negligible* probabilities were assigned, e.g. live pigs from Zones 1 and 2; insectivorous bats (all zones); fresh or frozen pork products (Zone 2) and processed pork products (Zones 1 & 2). However, it is important that this release assessment is revisited if imports of these commodities occur in the future, and especially if the imports are from Zone 1. Likewise, although imports did occur from all three zones, there is a high degree of uncertainty associated with the number of horses imported from Zone 3 due to the fact that only horses arriving from other EU MSs with a value greater than £260,000 are reported. In addition, the release assessment does not take into account events such as the Olympics 2012, when a larger numbers of horses will enter the UK. Therefore, *N* will increase for horses during this period, which may impact the overall probability of release.

The grouping of countries into zones greatly simplified this release assessment, whilst still identifying those countries that are of a higher likelihood of infection. However it is recognised that the classification of countries into zones will be very much impacted on by the under-reporting of outbreaks of henipaviruses within the Zones as no or low levels of surveillance (including lack of diagnostic capacity) in animal (fruit bats, horses and pigs) and human populations may result in henipavirus not being detected. This does provide notable uncertainty to the results of this release assessment. It is therefore important to review the epidemiological situation on a periodic basis and, if necessary, adjust the country groupings accordingly. Where possible, heterogeneity within the Zones has been taken into account, especially in relation to their past and current epidemiological situation and veterinary and public health services. If a greater level of certainty were required, a full assessment (country-by-country) could be undertaken, but this would be more time-intensive. However, this qualitative release assessment could be used as a screening tool, therefore highlighting the release routes and countries for which a more detailed assessment is required.

The validation of a release assessment such as this is problematic. For foodstuffs, testing was not undertaken, though if it was, the number of tests would be limited, so validation of the *Low* or *Very Low* probabilities of henipavirus contamination on foodstuffs would still be difficult. In the case of animals and humans, validation data is from the clinical surveillance of NiV and HeV. Companion animals, horses and humans entering the UK from Zone 1 are all assigned a *Very Low* probability of release. There have been no reported cases of NiV or HeV in the UK, hence suggesting that the assessed probabilities might be valid but does not inform us whether a *Negligible* or *Low* probability would actually be a better estimate. Therefore it is advised that more focus is placed on the relative results, that is between animal, humans and foodstuffs and, within these categories, between zones.

The consideration of the genus henipavirus, rather than NiV and HeV individually, may not be deemed appropriate for the animal release routes due to the inclusion of pigs and pig products and horses directly relating to the individual viruses NiV and HeV respectively. As yet, HeV has not been reported in pigs (only experimentally [35]) although NiV has been reported in horses in Malaysia [63]. The foodstuff release routes may be more impacted by any separation of HeV and NiV, mostly due to the differing amounts imported into the UK from the individual countries within Zones 1 & 2. However, since the level of importation from Zone 1 and Zone 2 countries is, in general, notably lower than Zone 3 it may be the case that the qualitative estimates of *N* will not change greatly if the Zones were to be redefined for the individual viruses HeV and NiV. Where possible, the heterogeneity between the different viruses and countries within the same zones has been considered when assigning and combining probabilties within this release assessment.

Because of the large number of release routes and the consideration of 3 zones for each route, a matrix approach was used to simplify the analysis. The use of a matrix to combine qualitative probabilities has both advantages and disadvantages. It provides a structured way of combining qualitative probabilities; thus increasing transparency to some extent. However there is no ‘true’ matrix, thus the matrix published by Gale *et al*, [41] was used but other examples include those given by Moutou *et al.* 2001 [64] and EFSA [65], which was also used by Heller *et al*. 2010 [66]. The choice of the matrix may influence the level of uncertainty as to the overall results of the release assessment. To assess the impact of adopting an alternative matrix, the EFSA matrix was also applied. The results (not shown) from this extra analysis showed that all of the overall release estimates (*R*) remained the same with the exception of bat bushmeat from zone 1, which was reduced from the original estimate of *Low* to *Very Low*.

Overall, this assessment assumes that fruit bats are the natural reservoir for henipaviruses, and that spill-over of henipaviruses to susceptible species is dependent on direct or indirect contact with an infected fruit bat e.g. via urine or contaminated fruit. Farming practices for the various countries where fruit bats are distributed are an important factor in determining whether humans or animals will become exposed to contaminated tissue or body fluids of infected bats. Further epidemiological and virological investigations need to be undertaken to address data gaps in the assessment such as the prevalence of henipaviruses in fruit bats, transmission of the virus, clinical signs and incubation period in species such as insectivorous bats, survival in different foodstuffs, etc. This release assessment could also be linked to an exposure and consequence assessment in order to estimate the overall risk to UK human and animal health.

## Supporting Information

Table S1 **Summary of import/movement data and results of the qualitative risk assessment.**
(DOCX)
