# Supplementary material for: Qualitative Release Assessment to Estimate the Likelihood of Henipavirus Entering the United Kingdom
Source: PLoS One. 2012 Feb 6;7(2):e27918. doi: 10.1371/journal.pone.0027918 (PMC3273481; doi:10.1371/journal.pone.0027918)
Supplement: Table S1 — Summary of import/movement data and results of the qualitative risk assessment. (DOCX) [file pone.0027918.s001.docx]

**Table S1**. Summary of import/movement data and results of the qualitative risk assessment.

|  | **Zone** | ***P_1_*** | ***P_2_*** | ***P_3_*** | ***P_4_*** | ***P_5_*** | ***P*** | ***N*** | ***R*** |
| --- | --- | --- | --- | --- | --- | --- | --- | --- | --- |
| ANIMALS/HUMANS | | | | | | | | | |
| Fruit bats | 1 | Medium | High | - | - | - | **Medium** | 0 | **Negligible** |
|  | 2 | Low | High | - | - | - | **Low** | 0 | **Negligible** |
|  | 3 | Very Low | High | - | - | - | **Very Low** | 22^(1)^ | **Negligible** |
| Insectivorous bats – import | 1 | Low | High | - | - | - | **Low** | 0 | **Negligible** |
|  | 2 | Very Low | High | - | - | - | **Very Low** | 0 | **Negligible** |
|  | 3 | Negligible | High | - | - | - | **Negligible** | 0 | **Negligible** |
| Insectivorous bats – flight | 3 | Negligible | - | High | - | - | **Negligible** | Very Low | **Negligible** |
| Companion animals | 1 | Very Low | Very Low | - | - | - | **Very Low** | 1765^(2)^ | **Very Low** |
|  | 2 | Negligible | Very Low | - | - | - | **Negligible** | 26 | **Negligible** |
|  | 3 | Negligible | Very Low | - | - | - | **Negligible** | 33834 | **Negligible** |
| Pigs | 1 | Low | Low | - | - | - | **Low** | 0 | **Negligible** |
|  | 2 | Very Low | Low | - | - | - | **Very Low** | 0 | **Negligible** |
|  | 3 | Negligible | Medium | - | - | - | **Negligible** | 438,725^(3)^ | **Negligible** |
| Horses | 1 | Low | High | - | - | - | **Low** | 86 | **Very Low** |
|  | 2 | Very Low | High | - | - | - | **Very Low** | 3 | **Negligible** |
|  | 3 | Negligible | High | - | - | - | **Negligible** | High^(4)^ | **Negligible** |
| Humans | 1 | Very Low | - | - | High | - | **Very Low** | Very High | **Very Low** |
|  | 2 | Negligible | - | - | High | - | **Negligible** | Very High | **Negligible** |
|  | 3 | Negligible | - | - | High | - | **Negligible** | Very High | **Negligible** |
| **FOODSTUFFS (TONNES)** | | | | | | | | | |
| Raw/frozen pork products | 1 | Low | - | - | - | Negligible | **Negligible** | 28^(5)^ | **Negligible** |
|  | 2 | Very Low | - | - | - | Negligible | **Negligible** | 0 | **Negligible** |
|  | 3 | Negligible | - | - | - | Medium | **Negligible** | 402,542 | **Negligible** |
| Processed pork products | 1 | Very Low | - | - | - | Negligible | **Negligible** | 0^(6)^ | **Negligible** |
|  | 2 | Negligible | - | - | - | Negligible | **Negligible** | 0^(6)^ | **Negligible** |
|  | 3 | Negligible | - | - | - | Medium | **Negligible** | 369,771^(6)^ | **Negligible** |
| Fruit | 1 | Low | - | - | - | Low | **Low** | 38,832^(7)^ | **Low** |
|  | 2 | Very Low | - | - | - | Low | **Very Low** | 117,823^(7)^ | **Low** |
|  | 3 | Negligible | - | - | - | Medium | **Negligible** | 2,310,452^(7)^ | **Negligible** |
| Fruit juice | 1 | Low | - | - | - | Negligible | **Negligible** | 743 | **Negligible** |
|  | 2 | Very Low | - | - | - | Negligible | **Negligible** | 10,209 | **Negligible** |
|  | 3 | Negligible | - | - | - | Medium | **Negligible** | 12,188 | **Negligible** |
| Bat bushmeat | 1 | High | - | - | - | High | **High** | Very Low | **Low** |
|  | 2 | Medium | - | - | - | High | **Medium** | Very Low | **Very Low** |
|  | 3 | Very Low | - | - | - | High | **Very Low** | Very Low | **Negligible** |

(1) 10 fruit bats imported from Mauritius (no species given); 12 red flying fox bats from New Zealand.

(2) 640 cats, 1125 dogs imported from Australia.

(3) EU only. Mostly pigs from Ireland

(4) 2876 horses were recorded as entering the UK from Zone 3. 293 were from 3^rd^ countries (mostly Dubai and New Zealand). 2583 horses were recorded as entering the UK from other EU MSs; however only horses above a £260,000 value are recorded. Therefore the risk was adjusted to take into account this under-reporting.

(5) All pork products imported from Australia.

(6) Pork products recorded under the category “Pig meat and offal (other than liver) prepared or preserved” were assumed to have received further processing.

(7) Includes fresh fruit, dried fruit, prepared fruit and preserved fruit.
